# NbHDR, A Host Protein Involved in the MEP Pathway, Interacts With *Bamboo Mosaic Virus* Replicase and Enhances Viral Accumulation

**DOI:** 10.1111/mpp.70099

**Published:** 2025-06-24

**Authors:** Chi Hzeng Wong, Chung‐Chi Hu, Ching‐Hsiu Tsai, Na‐Sheng Lin, Yau‐Heiu Hsu, Ming‐Kuem Lin, Ying‐Wen Huang

**Affiliations:** ^1^ Graduate Institute of Biotechnology National Chung Hsing University Taichung Taiwan; ^2^ Advanced Plant and Food Crop Biotechnology Center National Chung Hsing University Taichung Taiwan; ^3^ Institute of Plant and Microbial Biology Academia Sinica Taipei Taiwan; ^4^ Department of Chinese Pharmaceutical Sciences and Chinese Medicine Resources, College of Chinese Medicine China Medical University Taichung Taiwan

**Keywords:** 2‐C‐methyl‐D‐erythritol 4‐phosphate (MEP) pathway, bamboo mosaic virus (BaMV), HDR, replicase

## Abstract

Plant viruses, as obligate parasites, depend on host cellular machinery for various processes essential to their life cycle, making the investigation of these interactions fundamentally important. Bamboo mosaic virus (BaMV), a positive‐strand (+) RNA virus, serves as a model to explore host–virus interactions during replication. In this study, *Nicotiana benthamiana* 1‐hydroxy‐2‐methyl‐butenyl 4‐diphosphate reductase (NbHDR), a key enzyme in the methylerythritol 4‐phosphate (MEP) pathway, was identified as an interactor with BaMV replicase through immunoprecipitation, pull‐down and yeast two‐hybrid assays. Knockdown of *NbHDR* significantly reduced the accumulation of BaMV RNA and coat protein but did not affect infection with the close relative potato virus X, indicating its specific involvement in BaMV replication. Overexpression of *NbHDR* in *N. benthamiana* or the addition of NbHDR in in vitro RdRp reactions demonstrated that NbHDR enhances BaMV replication by promoting (+) RNA synthesis. To further explore whether the role of NbHDR in BaMV replication is linked to gibberellic acid (GA) synthesis through the MEP pathway, individual knockdowns of *NbHDR* and *ent‐kaurene synthase* (*KS*), a key enzyme in GA biosynthesis, were performed. Silencing *KS* sireduced BaMV accumulation, which was rescued by exogenous GA, but GA supplementation was insufficient to restore BaMV levels after *NbHDR* silencing. These findings suggest that NbHDR associates with the BaMV replication complex to enhance viral replication efficiency through a mechanism independent of GA synthesis from the MEP pathway, providing new insights into host–virus interactions.

## Graphical abstract

Chloroplast protein NbHDR directly interacts with BaMV replicase to enhance viral replication by promoting (+) RNA synthesis, independently of its role in gibberellic acid biosynthesis.

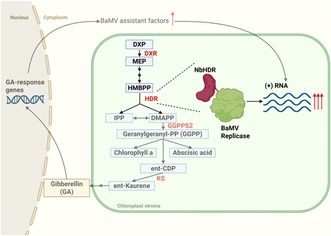

## 1 Introduction

Plant viruses, with their limited coding capacity and simple genetic composition, have developed effective strategies to become major pathogens in plants. They achieve this by recruiting various host proteins to facilitate critical processes, including replication, intracellular trafficking, cell‐to‐cell movement and systemic infection (Hyodo and Okuno 2020; Wang 2015). For positive‐strand (+) RNA viruses, replication is a highly coordinated process involving several distinct steps following the translation of the viral replicase: (i) recruitment of RNA templates by the replicase, (ii) transport of the replicase to the replication site, (iii) assembly of the viral replication complex (VRC) in association with host membranes and (iv) synthesis of negative‐strand (−) RNA intermediates, followed by the production of more progeny (+) RNA, with some viruses also generating subgenomic RNAs (sgRNAs) (Ahlquist et al. 2003; Ishibashi et al. 2012). Extensive molecular studies have identified numerous host factors involved in these replication steps, highlighting the critical roles of chloroplast‐associated proteins in promoting viral replication (Bhattacharyya and Chakraborty 2018; Budziszewska and Obrepalska‐Steplowska 2018; Bwalya and Kim 2023; Zhao et al. 2016).

The chloroplast plays a central role in plant cells by facilitating biological processes such as photosynthesis, which converts light energy into chemical energy to support plant growth (Lu and Yao 2018). In addition to its role in energy production, substantial evidence suggests that chloroplasts are involved in regulating plant immune responses by generating reactive oxygen species (ROS) and synthesising phytohormones and phenolic compounds, all of which contribute to distinct defence mechanisms (Krieger‐Liszkay et al. 2011; Straus et al. 2010). Phytohormones such as salicylic acid (SA), jasmonic acid (JA), gibberellic acid (GA) and abscisic acid (ABA) are critical for intracellular and intercellular signalling. Modulating the biosynthesis of these hormones can activate systemic acquired resistance, thereby enhancing defence against pathogen infections (Alazem and Lin 2015; Collum and Culver 2016; Santner and Estelle 2009; Zhao and Li 2021). However, many viruses target the chloroplast and manipulate its functions through protein–protein interactions, enabling them to promote viral replication and movement within host plants (Cheng et al. 2021; Kaido et al. 2014; Wei et al. 2013). Consequently, the chloroplast becomes a critical battleground during host–virus interactions (Yang et al. 2021).

*Bamboo mosaic* *virus* is a single‐stranded, positive‐sense (+) RNA virus classified under the genus *Potexvirus* in the family *Alphaflexiviridae* (Hsu et al. 2018). Its RNA genome is approximately 6.4 kb in length, featuring a 5′ cap structure and a 3′ poly(A) tail (Lin et al. 1994). The genome encodes five open reading frames (ORFs), each responsible for producing functionally distinct proteins. ORF1 encodes the viral replicase, which contains methyltransferase, helicase‐like, and RNA‐dependent RNA polymerase (RdRp) domains essential for replication (Meng and Lee 2017). ORFs 2–4 encode the triple gene block (TGB) proteins (TGBp1, TGBp2 and TGBp3), which facilitate viral movement between plant cells (Chou et al. 2013). ORF5 encodes the coat protein (CP), which plays roles in viral encapsidation, systemic movement and symptom development (Hung et al. 2014; Lan et al. 2010). Previous studies have identified several host factors that interact with bamboo mosaic virus (BaMV) to enhance viral accumulation (Huang et al. 2017). These host factors were identified using various approaches, including ultraviolet (UV) cross‐linking, co‐immunoprecipitation (Co‐IP), and cDNA‐amplified fragment length polymorphism (AFLP). These techniques were designed to screen for RNA‐binding proteins, protein–protein interactions, and differential gene expression in *Nicotiana benthamiana* during BaMV infection. For instance, UV cross‐linking identified interactions between viral RNA and chloroplast phosphoglycerate kinase (chlPGK) (Lin et al. 2007), heat shock protein 90 (Hsp90) (Huang et al. 2012), and photosystem II oxygen‐evolving complex protein (PsbO1) (Huang et al. 2021), which facilitate BaMV replication and transcription. Similarly, cDNA‐AFLP identified carbonic anhydrase (NbCA) (Chen et al. 2017), ferredoxin‐NADP+ oxidoreductase (FNR) (Chen et al. 2022), and non‐specific lipid transfer protein 1 (NbLTP1) (Chiu et al. 2020) as proviral factors. Additionally, the chloroplastic heat shock protein 70 (CPHsp70) was identified through Co‐IP assays as a positive regulator of BaMV replication (Huang, Hu, et al. 2017). Many of these host factors are associated with chloroplasts, emphasising the essential role of chloroplasts in BaMV replication. Their localisation within the chloroplast suggests that BaMV exploits chloroplast‐related pathways to optimise its replication efficiency. However, the molecular mechanisms and specific roles of chloroplast‐related proteins that interact with BaMV components and participate in BaMV replication remain to be fully elucidated.

In this study, we identified 1‐hydroxy‐2‐methyl‐butenyl 4‐diphosphate reductase (NbHDR) as an interactor with BaMV replicase in purified replicase complexes from BaMV‐infected *N. benthamiana*, using immunoprecipitation coupled with mass spectrometry (IP‐MS). Interaction assays confirmed that NbHDR directly interacts with BaMV replicase, and its localisation remains unchanged upon BaMV infection. Additionally, we demonstrated that NbHDR promotes RNA and CP accumulation of BaMV in infected cells. NbHDR is involved in the plastidial methylerythritol 4‐phosphate (MEP) pathway, which plays a key role in producing chlorophylls, terpenes, and GA (Banerjee and Sharkey 2014). Previous studies have shown that *N. benthamiana* 1‐deoxy‐d‐xylulose‐5‐phosphate reductoisomerase (NbDXR), an upstream enzyme in the MEP pathway, positively regulates BaMV accumulation (Huang et al. 2022). Silencing *NbDXR* significantly reduced BaMV accumulation, which was directly attributed to decreased GA production. However, our results revealed that exogenous GA supplementation was insufficient to restore BaMV accumulation after *NbHDR* silencing, suggesting that the effect of NbHDR on BaMV replication is independent of downstream GA synthesis. This study provides new insights into the role of chloroplast‐associated proteins, particularly NbHDR, in directly regulating BaMV replication.

## 2 Results

### 2.1 Identification of NbHDR in the BaMV Replication Complexes by IP‐MS

**FIGURE 1:**
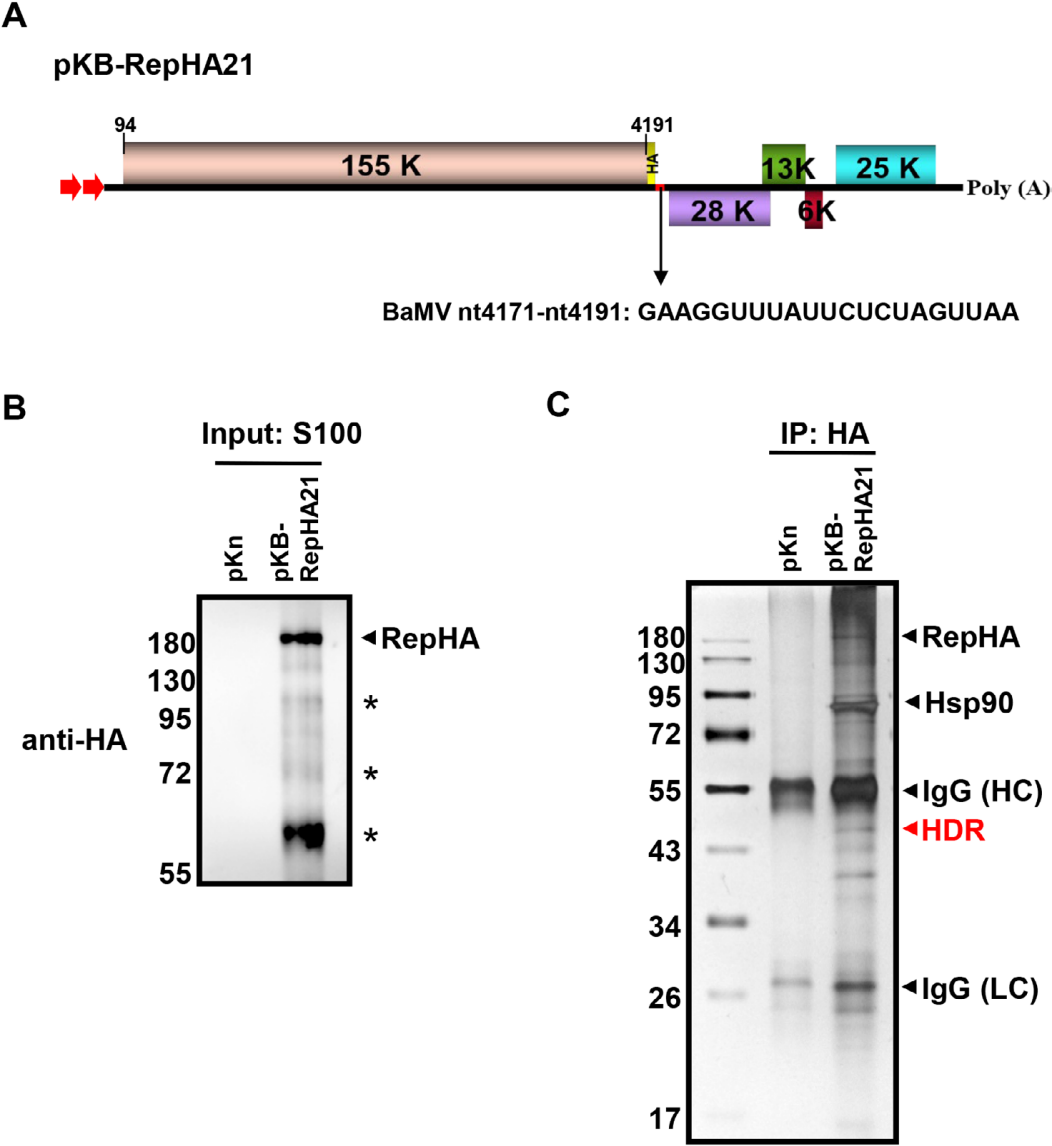
Identification of host proteins co‐purified with bamboo mosaic virus (BaMV) replicase. (A) Schematic representation of the genome organisation of BaMV expressing Rep‐HA (pKB‐RepHA21) under the control of a dual 35S promoter (red arrows). The BaMV genome encodes five open reading frames: the replicase (155K), triple gene block proteins (TGBp1‐3; 28, 13 and 6 K), and coat protein (CP; 25K). The red bar downstream of the HA tag in pKB‐RepHA21 denotes the 21‐nucleotide insertion restoring the putative TGB sgRNA promoter sequence, shown below the panel. (B) Western blot analysis of BaMV replicase isolated from leaves infiltrated with an empty vector (pKn) or pKB‐RepHA21 using an anti‐HA antibody. The arrowhead indicates the position of Rep‐HA, and presumed degraded forms of Rep‐HA are marked with asterisks (*). (C) Co‐purified proteins from anti‐HA immunoprecipitation (IP) were separated by SDS‐PAGE and visualised by silver staining. Protein bands of interest, including some identified based on previous study, were excised and analysed via liquid chromatography–tandem mass spectrometry (LC–MS/MS).

In our previous study, we constructed an infectious clone of BaMV, designated as pKBRepHA21, which contains an HA‐tagged replicase (Figure 1A). To ensure proper viral function, the core region of the BaMV sgRNA1 promoter was inserted downstream of the HA tag, allowing the restoration of sgRNA1 transcription (Huang, Hu, et al. 2017). This modification not only preserved the functionality of BaMV replication and transcription but also enabled efficient immunoprecipitation of the replicase, facilitating the identification and characterisation of host factors associated with the BaMV replication complexes.

To investigate the host proteins associated with the BaMV replication, we purified BaMV replicase from *N. benthamiana* leaves infiltrated with *Agrobacterium* carrying empty vector (EV, pKn) or pKB‐RepHA21. The detergent‐solubilised membrane fractions were precleaned with protein A‐Sepharose to produce input samples. BaMV Rep‐HA was successfully detected in inputs from leaves infiltrated with pKBRepHA21 but not in those infiltrated with the EV, demonstrating that complexes containing Rep‐HA can be specifically captured using an anti‐HA antibody (Figure 1B). The purified complexes were subjected to immunoprecipitation with an anti‐HA antibody bound to protein A beads, followed by SDS‐PAGE with silver staining (Figure 1C). Unique bands of 43–55 and 72–130 kDa were observed in the BaMV‐infected samples, and these regions were excised and analysed by liquid chromatography–tandem mass spectrometry (LC–MS/MS). The analysis identified NbHDR as one of the host proteins with high sequence coverage (46%) and a molecular weight consistent with its expected size (Figure S1a). These findings suggest that NbHDR is a potential host factor associated with the BaMV replication complexes.

### 2.2 Effect of BaMV Infection on *NbHDR* Expression and Subcellular Localisation

NbHDR is a nuclear‐encoded chloroplast protein with an approximate molecular weight of 52 kDa, featuring a 66‐amino acid chloroplast transit peptide at its N‐terminus, as predicted by ChloroP (Emanuelsson et al. 1999). Upon chloroplast import, this transit peptide is cleaved, yielding the mature form of NbHDR with a lower molecular weight. As a key enzyme in the MEP pathway, NbHDR catalyses the reduction of (*E*)‐4‐hydroxy‐3‐methyl‐but‐2‐enyl diphosphate (HMBPP) to isopentenyl diphosphate (IPP) and dimethylallyl diphosphate (DMAPP), which are essential precursors for isoprenoid biosynthesis (Hsieh and Hsieh 2015).

**FIGURE 2:**
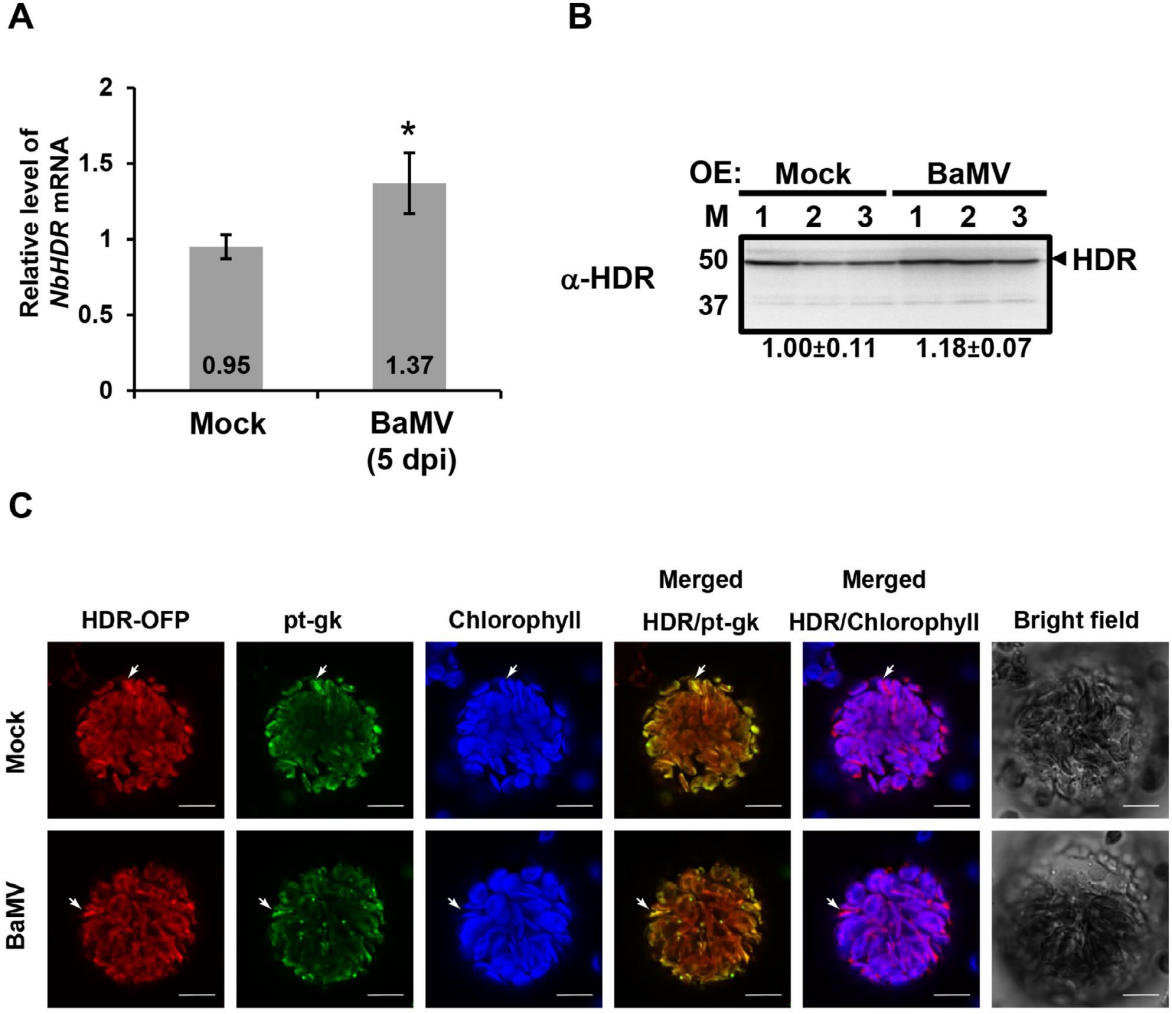
Effect of bamboo mosaic virus (BaMV) infection on NbHDR accumulation and subcellular localisation. (A) The relative gene expression levels of *NbHDR* in mock‐ and BaMV‐infected plants were measured by reverse transcription‐quantitative PCR (RT‐qPCR) at 5 days post‐inoculation (dpi). Data represent the mean ± SD obtained from three independent experiments, with four individual plants for each experiment (Student's *t* test: *p < 0.05). (B) Western blot analysis of NbHDR protein levels in mock and BaMV‐infected plants. Total proteins were extracted at 5 dpi, and NbHDR was detected using antiserum against NbHDR. Coomassie blue staining of the gel served as the loading control. (C) Subcellular localisation of HDR‐OFP in *Nicotiana benthamiana* protoplasts co‐expressing pt‐gk, a stroma marker, with or without BaMV infection at 3 dpi. Confocal microscopy images show HDR‐OFP localised in the chloroplast stroma, as indicated by its colocalisation with pt‐gk fluorescence. White arrows highlight regions where NbHDR‐OFP signals overlap with pt‐gk while remaining distinct from chlorophyll autofluorescence, confirming its stromal localisation. Scale bars: 10 μm.

To investigate the impact of BaMV infection on *NbHDR* expression, we analysed NbHDR mRNA and protein levels in *N. benthamiana* leaves at 5 days post‐inoculation (dpi) of BaMV using reverse transcription‐quantitative PCR (RT‐qPCR) and western blotting. The results showed a slight increase in both NbHDR mRNA and protein accumulation in BaMV‐infected samples compared to the mock inoculation, with fold changes of approximately 1.4 and 1.2, respectively (Figure 2A,B).

To determine whether BaMV infection affects the subcellular localisation of NbHDR, we co‐infiltrated *N. benthamiana* leaves with a chloroplast stroma marker, pt‐gk (Nelson et al. 2007), and NbHDR fused to OFP (HDR‐OFP), with or without BaMV infection. Confocal microscopy of protoplasts revealed that NbHDR fluorescence strongly colocalised with the stroma marker (pt‐gk), confirming its stromal localisation (Figure 2C, arrows). Additionally, NbHDR fluorescence was spatially distinct from the thylakoid‐associated chlorophyll autofluorescence, further supporting its localisation in the chloroplast stroma. This distribution pattern remained unchanged upon BaMV infection, indicating that viral infection does not alter NbHDR subcellular localisation (Figure 2C).

### 2.3 BaMV Replicase Interacts With NbHDR Through the RdRp Domain

**FIGURE 3:**
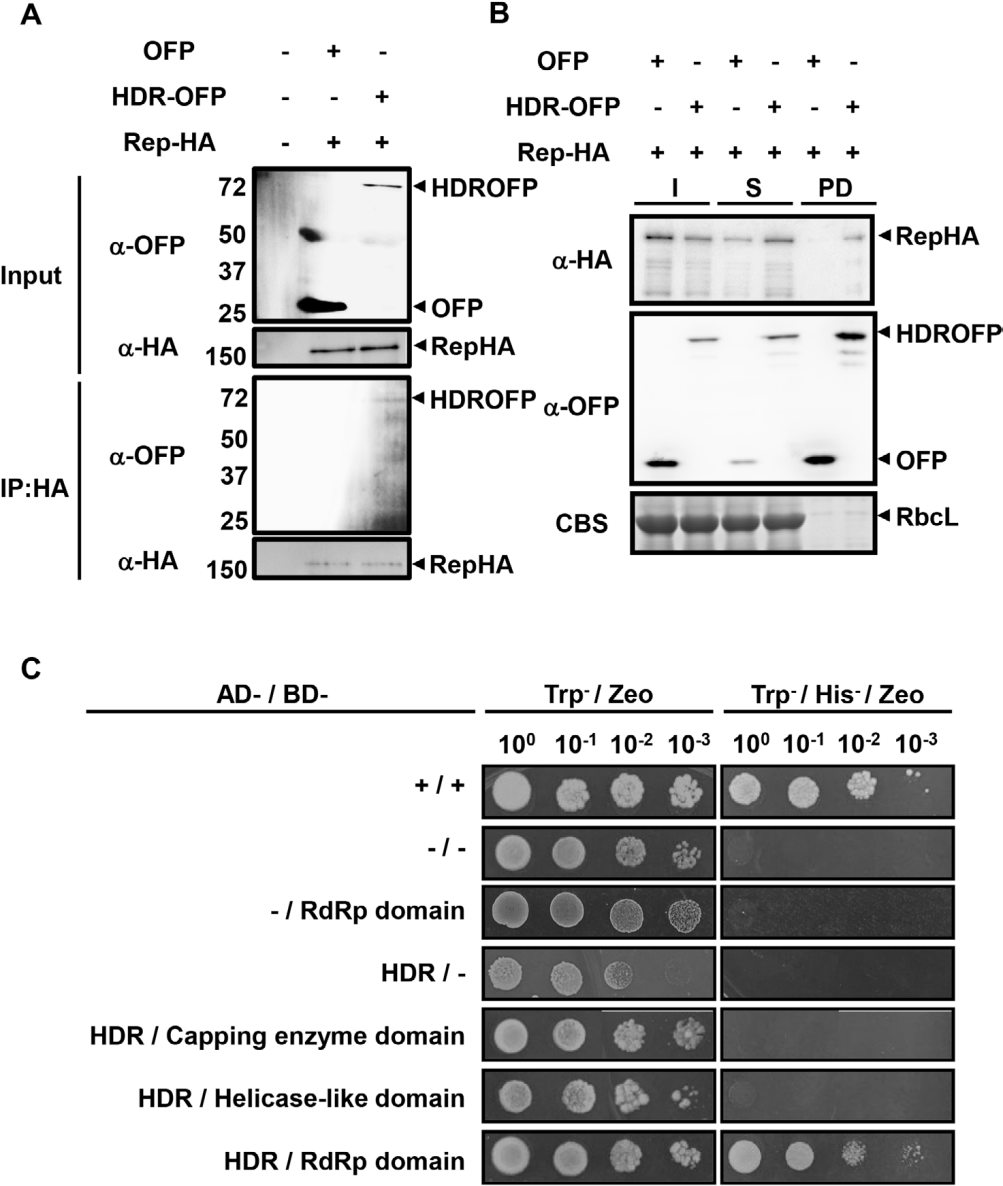
Interaction between NbHDR and bamboo mosaic virus (BaMV) replicase. (A) Co‐immunoprecipitation (Co‐IP) assay to detect the interaction between replicase‐HA and HDR‐OFP. Crude extracts were prepared from *Nicotiana benthamiana* leaves infiltrated with *Agrobacterium* carrying pEOFP and pERepHA21 or pEHDR‐OFP and pERepHA21, with healthy leaves serving as a control. Total proteins were incubated with anti‐HA antibody followed protein A beads for immunoprecipitation. Western blot analysis was performed using antisera against OFP and HA to detect co‐immunoprecipitated proteins. (B) Pull‐down assay to verify the interaction between Replicase‐HA and HDR‐OFP using anti‐RFP magnetic beads. Crude extracts from infiltrated leaves at 3 days after infiltration were incubated with RFP‐trap magnetic beads. The input (I; total protein), supernatant (S; unbound proteins) and pull‐down (PD; precipitated proteins) fractions were analysed by SDS‐PAGE and western blotting with antisera against OFP and HA. (C) Yeast two‐hybrid (Y2H) assay to test the interaction between NbHDR and BaMV replicase domains. The BaMV replicase was divided into three domains—capping enzyme, helicase‐like, and RdRp—and fused to the Gal4 DNA‐binding domain (BD), while NbHDR was fused to the Gal4 activation domain (AD). Transformed *Saccharomyces cerevisiae* L40 cells were plated on control medium (−Trp/Zeocin) and selective medium (−Trp/−His/Zeocin). Positive control (+/+) and negative control (−/−) were included. The yeast concentrations along with their dilution ratios are indicated at the top of each panel.

To further explore the interaction between NbHDR and BaMV replicase and better understand their relationship, we examined their interaction in the absence of other viral components. For this, *N. benthamiana* leaves were co‐infiltrated with constructs expressing either HDR‐OFP and Rep‐HA or OFP and Rep‐HA, and Co‐IP was performed. Total proteins were extracted and immunoprecipitated using an anti‐HA antibody. The results showed that NbHDR‐OFP specifically interacted with Rep‐HA, while OFP alone did not (Figure 3A).

Additionally, an RFP pull‐down assay using RFP‐trap protein‐conjugated beads, which recognise OFP, was conducted. The results demonstrated that NbHDR‐OFP, but not OFP alone, successfully co‐precipitated Rep‐HA (Figure 3B). These findings indicate that the interaction between NbHDR and BaMV replicase occurs independently of other viral proteins or viral RNA.

To further validate these results, we performed yeast two‐hybrid (Y2H) assays. The three functional domains of BaMV replicase—the capping enzyme domain, helicase‐like domain, and RdRp core domain—were individually fused to the Gal4 DNA‐binding domain (BD). NbHDR was fused to the Gal4 activation domain (AD). A positive interaction between the BD and AD fusions activates *His3* expression in yeast, allowing colony formation on plates lacking histidine (−Trp/−His/−Zeo). Plates containing zeocin in the absence of tryptophan (−Trp/Zeo) were used to confirm successful plasmid transformation. Yeast cells expressing BD‐Fos2 and AD‐Jun served as positive controls (+/+), while combinations of empty vectors (BD plus AD, −/−) or negative control combinations [(BD‐RdRp plus AD) and (BD plus AD‐HDR)] excluded nonspecific activation. The Y2H results showed that the RdRp domain of BaMV replicase (BD‐RdRp) interacted with NbHDR (AD‐HDR), while no interactions were observed for the capping enzyme domain or the helicase‐like domain (Figure 3C). These findings collectively demonstrate that NbHDR interacts with BaMV replicase both in vivo and in vitro, probably through the RdRp domain of the replicase.

### 2.4 Downregulation of *NbHDR* Reduces BaMV Accumulation in *N. benthamiana* Leaves and Protoplasts

**FIGURE 4:**
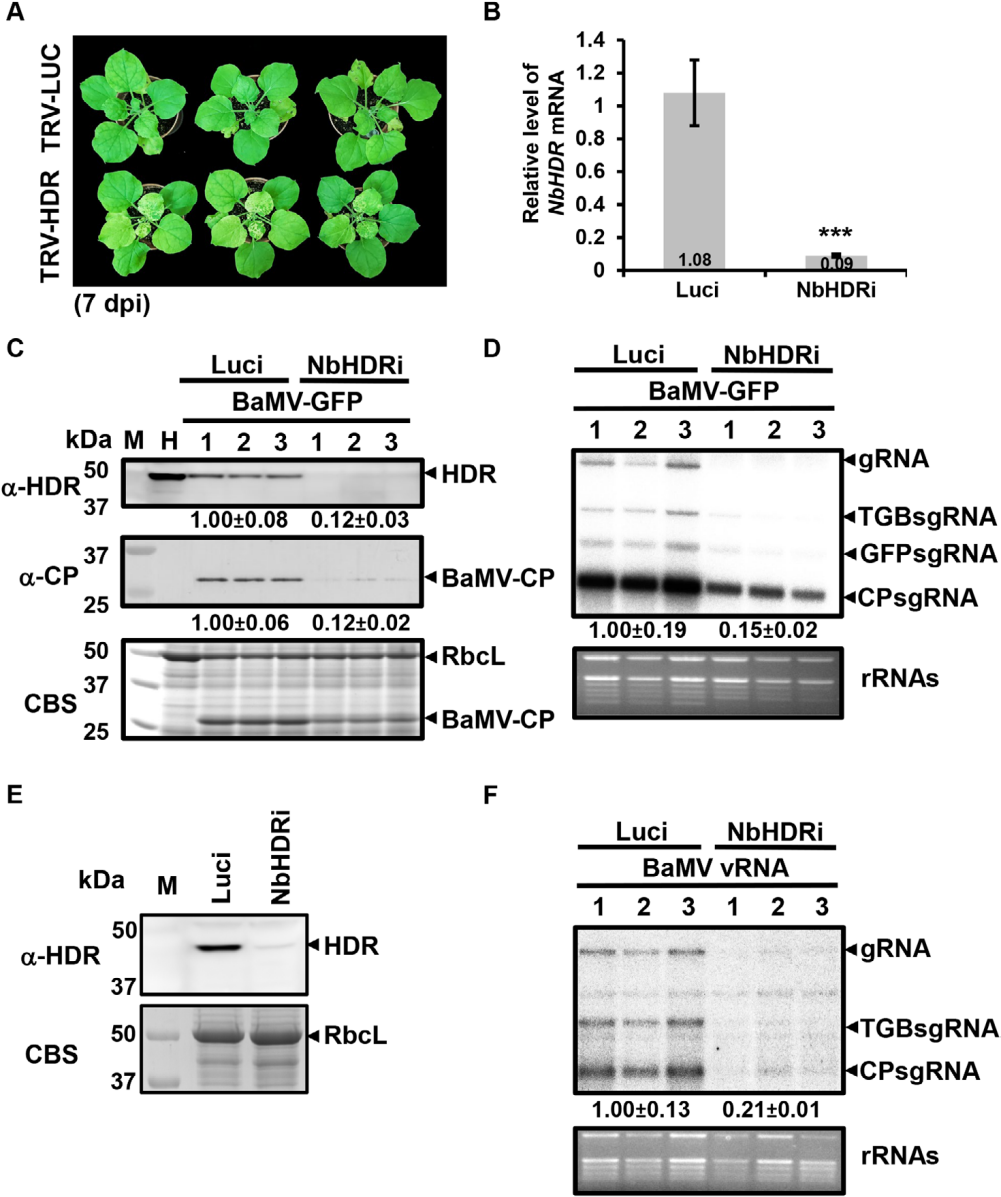
Effect of *NbHDR* knockdown on bamboo mosaic virus (BaMV) accumulation in *Nicotiana benthamiana* plants and protoplasts. (A) Phenotype of *Luc*‐silenced (TRV‐Luc, control) and *NbHDR*‐knockdown (TRV‐HDR) *N. benthamiana* plants at 7 days post‐inoculation (dpi). (B) Relative *NbHDR* gene expression levels in control (Luci) and *NbHDR*‐knockdown plants (NbHDRi) were quantified by reverse transcription‐quantitative PCR at 7 dpi. Data represent the mean ± SD obtained from three independent experiments, with four individual plants for each experiment (Student's *t* test: ***p < 0.001). (C, D) Western and northern blot analyses of BaMV accumulation in virus‐induced gene silencing (VIGS) plants. At 7 dpi, the upper leaves of control and silenced plants were infiltrated with *Agrobacterium* carrying pKBG (BaMV‐GFP). Total RNA and proteins were extracted at 3 dpi. H represents the healthy plant control. Western blot analysis was performed with antiserum against BaMV coat protein (CP), and northern blot analysis was used to detect BaMV genomic RNA (gRNA) and subgenomic RNAs (sgRNAs). (E) Western blot analysis of NbHDR protein levels in Luci and NbHDRi plants. At 7 dpi, protoplasts were isolated from the upper leaves and inoculated with BaMV viral RNA. Western blot detection of NbHDR was performed with antiserum against NbHDR. Coomassie blue staining was used as a loading control. (F) Northern blot analysis of BaMV RNA accumulation in Luci and NbHDRi protoplasts at 1 dpi. BaMV gRNA and sgRNAs were detected, with EtBr‐stained rRNA shown as the loading control.

To investigate the role of NbHDR in BaMV infection, we used a tobacco rattle virus (TRV)‐based virus‐induced gene silencing (VIGS) system. The 3′ untranslated region (UTR) of *NbHDR* was amplified and cloned into the TRV2 vector to generate TRV2‐HDR. *N. benthamiana* leaves were co‐infiltrated with TRV1 and TRV2‐HDR or with TRV1 and TRV2‐Luc (silencing of the luciferase gene) as the mock control. At 10 dpi, *NbHDR*‐silenced plants exhibited severe photobleaching in the upper leaves (Figure S2), probably resulting from the reduced expression of *HDR*, an essential upstream gene for chlorophyll biosynthesis. However, the bleaching could affect the physiological processes of *N. benthamiana*, making it unsuitable for further infection studies. To avoid this, we optimised the conditions by performing subsequent experiments at 7 dpi, when the silencing effect was established without severely affecting plant physiological status (Figure 4A). At 7 dpi, RT‐qPCR analysis revealed a significant reduction in *NbHDR* mRNA levels to 9% of the control (Figure 4B). Western blotting further confirmed a substantial decrease in NbHDR protein accumulation (Figure 4C, upper panel). Mild chlorosis was observed in the upper leaves of *NbHDR*‐silenced plants, while *Luc*‐silenced control plants appeared phenotypically normal (Figure 4A).

To investigate the effect of *NbHDR* knockdown on BaMV infection, we used agroinfiltration to introduce pKBG (Prasanth et al. 2011), a BaMV infectious clone expressing GFP, into *NbHDR*‐silenced *N. benthamiana* leaves. GFP fluorescence provided a convenient method for monitoring viral accumulation and spread, while viral accumulation was assessed by western and northern blot analyses. Western blot analysis confirmed a significant reduction of NbHDR protein levels in silenced plants at 3 dpi (Figure 4C). BaMV CP and genomic RNA (gRNA) levels were significantly reduced to 12% and 15%, respectively, compared to the control (Figure 4C,D). To assess whether *NbHDR* silencing affects other potexviruses, *N. benthamiana* plants silenced for *NbHDR* were inoculated with potato virus X (PVX). Silencing of *NbHDR* did not affect PVX infection, as neither PVX CP nor viral gRNA levels were altered compared to the control (Figure S3). These findings suggest that knockdown of *NbHDR* specifically impacts BaMV accumulation without causing nonspecific effects on viral infection.

To further elucidate how NbHDR affects BaMV accumulation, protoplasts were isolated from *Luc*‐ and *NbHDR*‐silenced *N. benthamiana* plants and inoculated with BaMV viral RNA. Western blot analysis confirmed a significant decrease in NbHDR protein levels in silenced protoplasts (Figure 4E). Correspondingly, the accumulation level of BaMV gRNA was significantly reduced to 21% of that in control cells at 24 h post‐inoculation (hpi) (Figure 4F). These results indicate that NbHDR primarily contributes to BaMV replication within single cells, rather than influencing viral cell‐to‐cell movement.

### 2.5 Overexpression of *NbHDR* Upregulates BaMV Accumulation

**FIGURE 5:**
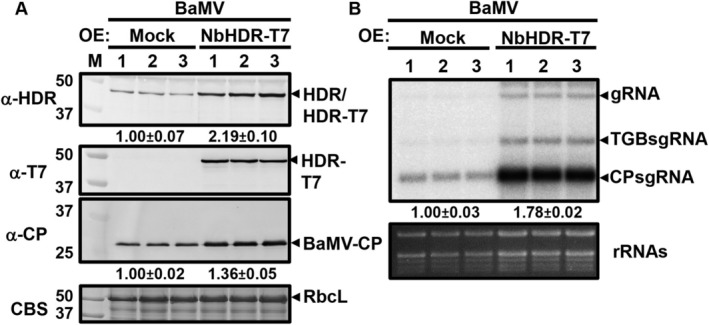
Effect of transient expression of *NbHDR* on bamboo mosaic virus (BaMV) accumulation. (A) Western blot analyses of BaMV accumulation in control (mock) and *NbHDR*‐overexpressing plants. Western blotting was performed using antisera against NbHDR, T7, and BaMV CP. Coomassie blue staining (CBS) was used as the loading control. (B) Northern blot analysis of BaMV viral RNA accumulation. α ^32^P‐labelled RNA probe was used to detect BaMV genomic RNA (gRNA) and subgenomic RNAs (sgRNAs). Data represent the mean ± SD obtained from three independent experiments, with three individual plants for each experiment. BaMV genomic RNA was quantified and normalised using 28S rRNA as the loading control.

To demonstrate the positive regulatory role of NbHDR in BaMV accumulation, we constructed a vector containing the full‐length coding sequence of NbHDR fused with a T7 tag at the C‐terminus, designated as pENbHDR‐T7. The role of NbHDR in BaMV accumulation was validated by analysis of BaMV RNA accumulation in *N. benthamiana* leaves co‐infiltrated with NbHDR‐T7 and BaMV at 3 days post‐agroinfiltration (dpai). Western blot analysis using an anti‐NbHDR antibody confirmed the expression of transiently overexpressed NbHDR‐T7 protein (Figure 5A). Compared to the control, the expression level of NbHDR‐T7 was 2.19‐fold higher, and this overexpression significantly enhanced BaMV accumulation. Specifically, BaMV CP and viral gRNA levels were 1.36‐fold and 1.78‐fold higher, respectively, in *NbHDR*‐overexpressing plants (Figure 5A,B). Taken together, the results from both silencing and overexpression experiments suggest that NbHDR positively regulates BaMV accumulation, highlighting its critical role in supporting viral replication and accumulation.

### 2.6 NbHDR Participates in BaMV Replication Independently of Gibberellic Acid Biosynthesis

**FIGURE 6:**
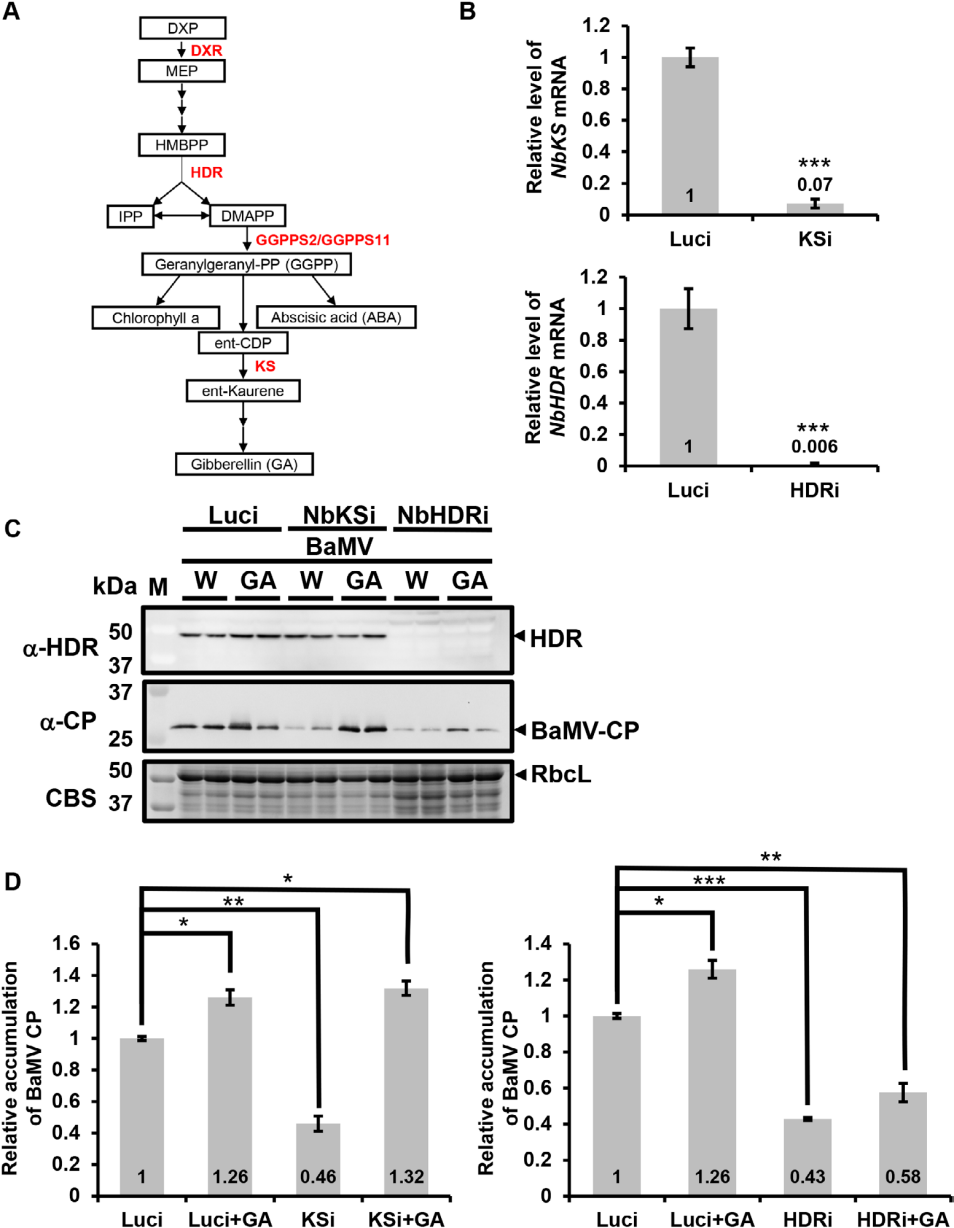
Effects of gibberellic acid (GA) treatment on bamboo mosaic virus (BaMV) accumulation in *NbKS* and *NbHDR* knockdown plants. (A) Schematic representation of the chloroplast methylerythritol 4‐phosphate (MEP) pathway, emphasising its role in GA‐related metabolism. Enzymes highlighted in red indicate those evaluated by virus‐induced gene silencing (VIGS) for their involvement in BaMV infection (Huang et al. 2022). (B) Relative gene expression levels of *NbKS* and *NbHDR* in control (Luci), KSi and HDRi plants at 7 days post‐infiltration. The mRNA levels were quantified by reverse transcription‐quantitative PCR, and data are presented as mean ± SD from three independent experiments, with four individual plants for each experiment. (C) Western blot analysis of BaMV accumulation in Luci, KSi and HDRi plants. GA (200 μM) or water (W, as control) was sprayed on the upper leaves 12 h before BaMV inoculation. Total proteins were extracted at 3 dpi. Western blotting was performed with antisera against NbHDR and BaMV coat protein (CP). Coomassie blue staining (CBS) of total protein served as the loading control. (D) Quantification of BaMV CP levels from panel (C). Data represent the mean ± SD obtained from three independent experiments, with two individual plants for each experiment (Student's *t* test: ***p < 0.001).

In a previous study (Huang et al. 2022), VIGS was systematically used to assess the roles of key enzymes in the plastidial MEP pathway on BaMV accumulation (Figure 6A). Several host factors, including NbDXR, NbGGPPS11, NbGGPPS2 and NbKS, have been examined. Notably, the knockdown of *NbDXR* and *NbKS* resulted in a significant reduction in BaMV accumulation, primarily due to their roles in gibberellic acid (GA) synthesis. NbHDR catalyses the synthesis of IPP and DMAPP, which are precursors for the synthesis of geranylgeranyl diphosphate (GGPP), an upstream metabolite of GA (Hedden 2020) (Figure 6A). In contrast, the present study demonstrated that NbHDR directly interacts with the BaMV replicase, suggesting that it may directly facilitate viral replication. To determine whether NbHDR promotes BaMV replication directly or through its involvement in GA biosynthesis, *NbHDR* and *NbKS* were separately silenced in *N. benthamiana*, followed by exogenous GA application and BaMV infection. GA treatment was applied to *N. benthamiana* plants at 7 days post‐silencing by spraying a 200 μM GA solution onto the leaves. BaMV virions were then inoculated 12 h post‐GA treatment. Total protein was extracted from the BaMV‐inoculated leaves at 3 dpi for analysis.

Silencing efficiency was confirmed by RT‐qPCR, showing significant reductions in *NbKS* and *NbHDR* mRNA levels to 7% and 0.6% of the control, respectively (Figure 6B). Both silencing treatments significantly reduced BaMV accumulation, with *NbKS* and *NbHDR* silencing decreasing CP levels to 46% and 43% of those in control plants, respectively. Notably, exogenous GA supplementation restored BaMV CP accumulation to basal levels in *NbKS*‐silenced plants, while it failed to rescue BaMV CP accumulation in *NbHDR*‐silenced plants (Figure 6C,D).

These findings suggest that NbHDR contributes to BaMV replication independently of its role in the MEP pathway for GA biosynthesis. Instead, NbHDR may play a direct role in facilitating BaMV replication.

### 2.7 NbHDR Enhances BaMV Replication by Facilitating (+) RNA Synthesis

Previous results demonstrated that NbHDR interacts with BaMV replicase and promotes BaMV accumulation in single cells independently of GA biosynthesis. To further confirm the direct role of NbHDR in BaMV replication, we performed an in vitro RdRp assay using detergent‐solubilised membrane fractions purified from BaMV‐infected *N. benthamiana* leaves. The assay measured the activity of BaMV replicase by incorporating radiolabelled UTP into newly synthesised viral RNA.

**FIGURE 7:**
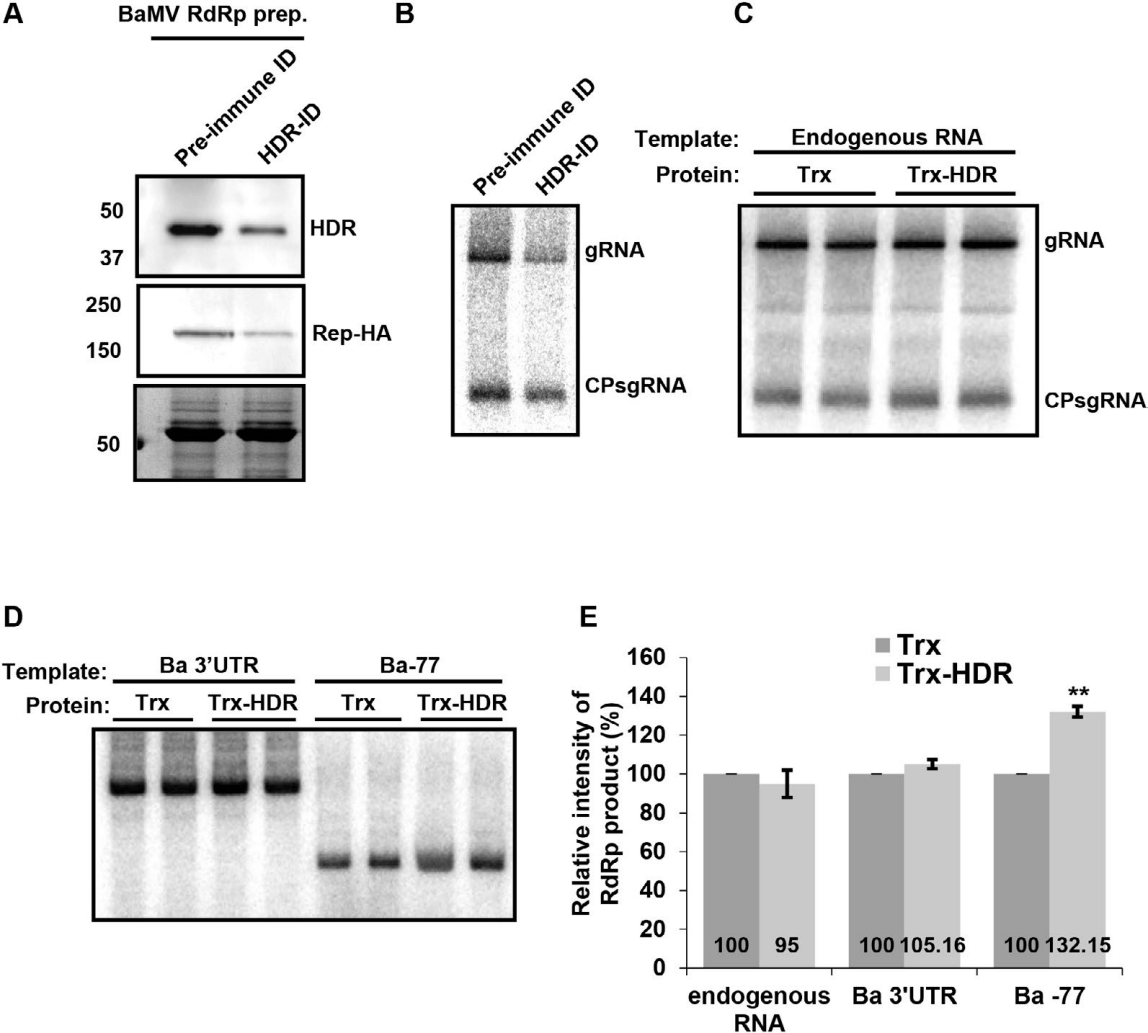
Effect of NbHDR depletion or addition on bamboo mosaic virus (BaMV) RNA synthesis in vitro. (A) Immunodepletion (ID) of NbHDR from BaMV RdRp preparations using pre‐immune or anti‐NbHDR antibodies. Depleted samples were analysed by western blotting with antisera against NbHDR and HA, with Coomassie blue staining used as a loading control. (B) In vitro RNA synthesis assay with BaMV RdRp complexes following ID. RNA products synthesised from endogenous templates were analysed by agarose gel electrophoresis and autoradiography. (C) Analysis of complementary strand RNA synthesis using endogenous RNA templates in the presence of Trx‐HDR (500 ng) or Trx (500 ng) proteins as controls. (D, E) In vitro RNA synthesis assay with exogenous RNA templates Ba 3′ untranslated region (UTR) and Ba‐77. RNA products were analysed as described above. Quantified data are presented as mean ± SD from three independent experiments, with two individual reactions for each experiment (**p < 0.01; Student's *t* test).

To test the effect of NbHDR depletion, immunodepletion (ID) was performed using anti‐NbHDR antibody to remove NbHDR from the replication complexes. Western blot analysis revealed that depleting NbHDR also significantly reduced BaMV replicase levels, confirming the interaction between NbHDR and the replicase. This finding demonstrates that the immunodepletion of NbHDR simultaneously removes BaMV replicase due to their physical association (Figure 7A). The immunodepleted samples were then subjected to in vitro RdRp assays, with ID using pre‐immune antisera serving as the control. The depletion of NbHDR caused a marked decrease in endogenous BaMV RNA synthesis (Figure 7B), highlighting the critical role of NbHDR in BaMV replication, although the reduction in BaMV replicase itself may also contribute to the decreased activity.

To determine which stage of replication NbHDR influences, we performed in vitro RdRp assays with recombinant NbHDR proteins. The HDR proteins were expressed in *Escherichia coli* as thioredoxin (Trx) fusions to enhance solubility (Figure S4), and purified Trx‐HDR was added to the reactions, with Trx alone serving as the control. Because BaMV replication complexes consist of endogenous viral RNA templates bound to replicase, assays using these complexes mainly reflect elongation activity for endogenous RNAs. To specifically assess the ability of replicase to recognise and initiate RNA synthesis on a new template, we pretreated the detergent‐solubilised membrane fractions with micrococcal nuclease to degrade the endogenous RNA. This allowed the replicase, originally occupied with endogenous RNA, to bind and initiate synthesis on exogenously added RNA templates. The promoter regions for BaMV (−) RNA and (+) RNA synthesis have been identified as the 3′ untranslated region (3′ UTR) of (+) RNA (Ba 3′ UTR) and the 3′‐terminal 77 nucleotides of (−) RNA (Ba‐77), respectively (Cheng and Tsai 1999; Lin et al. 2005). These exogenous templates were used to evaluate the role of NbHDR in viral replication.

When endogenous viral RNAs were used as the templates for complementary strand synthesis, the addition of NbHDR did not alter the elongation activity of BaMV replicase, indicating that NbHDR is not directly involved in the elongation phase (Figure 7C). However, when endogenous RNA was removed using micrococcal nuclease and exogenous RNA templates were provided, the addition of NbHDR significantly enhanced the initiation efficiency of (+) RNA synthesis using Ba‐77 as the template (Figure 7D,E). These findings suggest that NbHDR facilitates the initiation of (+) RNA synthesis, potentially by assisting BaMV replicase in recognising the (−) RNA template or assembling the complex for RNA replication initiation on Ba‐77.

## 3 Discussion

This study demonstrates that NbHDR plays a pivotal role in facilitating BaMV replication. By directly interacting with the BaMV replicase, NbHDR enhances the efficiency of (+) RNA synthesis, independently of its role in GA biosynthesis. Knockdown of *NbHDR* specifically reduced BaMV accumulation in single cells without affecting a closely related virus, PVX, suggesting a highly specific involvement in BaMV replication. Moreover, immunodepletion and in vitro RdRp assays confirmed that NbHDR is a key host factor in the formation and activity of the VRC. These findings uncover a novel function of NbHDR in viral replication, advancing our understanding of host‐virus interactions and highlighting the importance of host factors in supporting viral RNA synthesis.

Previous studies have demonstrated that the plastidial MEP pathway contributes to BaMV replication primarily through its downstream product, GA (Huang et al. 2022). GA has been shown to upregulate the expression of nuclear‐encoded chloroplast proteins, such as ferredoxin‐NADP^+^ reductase and carbonic anhydrase, which subsequently enhance BaMV accumulation (Chen et al. 2017; Chen et al. 2022). This suggests that GA plays a regulatory role in facilitating BaMV replication. In the present study, we examined the role of NbHDR, a key enzyme in the MEP pathway that catalyses the synthesis of IPP and DMAPP. Our findings revealed that the knockdown of NbHDR led to a significant reduction in BaMV accumulation, and this effect could not be rescued by exogenous GA supplementation (Figure 6). In contrast, silencing of NbKS—an enzyme specifically involved in GA biosynthesis—resulted in reduced BaMV levels that were restored upon GA application. This distinction indicates that NbHDR contributes to BaMV replication via a GA‐independent mechanism. To further dissect this mechanism, we conducted overexpression experiments using both NbHDR and a catalytically inactive NbHDR mutant (mNbHDR) carrying four point mutations (H236A, E238A, S374A and N376A; Gräwert et al. 2010). Both NbHDR and mNbHDR enhanced BaMV RNA accumulation, suggesting that NbHDR supports BaMV replication independently of its enzymatic activity (Figure S5). However, the replication‐promoting effect of mNbHDR was weaker than that of NbHDR, implying that while the physical interaction between NbHDR and the BaMV replicase is sufficient to enhance replication, its catalytic function and, by extension, MEP pathway‐derived metabolites also contribute to optimal viral replication. As a key enzyme producing IPP and DMAPP, NbHDR could influence other branches of isoprenoid metabolism, including steroid biosynthesis (Seemann et al. 2006). Steroids have been implicated in stabilising membrane structures and supporting replication compartment formation in other RNA viruses (Tarkowska and Strnad 2018). BaMV may similarly recruit host‐derived steroids or isoprenoid intermediates to assist in the assembly and stability of its VRCs. Therefore, our data suggest that GA biosynthesis is not the sole MEP pathway‐mediated process involved in BaMV replication and that NbHDR exerts dual roles—structural via direct interaction with replicase, and metabolic via downstream products—in supporting BaMV accumulation.

The chloroplast plays a dual role in plant–virus interactions, serving both as a site for viral replication and as a key hub for synthesising antiviral molecules, such as ROS and phytohormones, that regulate host defences (Bwalya and Kim 2023; Liu et al. 2024; Yang et al. 2021). In response, viruses have evolved mechanisms to evade or suppress these defences by targeting chloroplast‐associated proteins (Balasubramaniam et al. 2014; Gnanasekaran et al. 2019; Lehto et al. 2003; Zhai et al. 2021). Conversely, some chloroplast‐associated proteins are directly recruited to facilitate viral replication. For example, the nuclear inclusion protein b of *Tobacco vein banding mosaic virus* interacts with NbRPL1, a chloroplast ribosomal protein, to enhance viral infection (Cheng et al. 2021). Although direct visualisation of the BaMV replication protein in the chloroplast remains challenging due to its low expression levels, accumulating evidence suggests that BaMV replication is closely associated with the chloroplast stroma. For instance, chloroplast‐resident proteins such as cpHsp70 and NbFNR have been shown to interact with the BaMV replicase, and their proper localisation in the chloroplast is essential for efficient viral accumulation (Huang, Hu, et al. 2017; Chen et al. 2022). Moreover, NbPsbO1, a chloroplast protein normally associated with the thylakoid membrane, has been observed to translocate to the chloroplast stroma upon BaMV infection and specifically interact with the viral sgRNA promoter (Huang et al. 2021). Additionally, nuclear‐encoded chlPGK has been implicated in guiding viral RNA and associated proteins into the chloroplast stroma for replication (Lin et al. 2007). Our current findings further support this model, as NbHDR, a chloroplast stroma‐localised protein, was found to directly interact with the BaMV replicase (Figure 3). Collectively, these observations strongly suggest that the BaMV replicase is localised, at least in part, within the chloroplast stroma, where it forms active VRC.

The formation of a functional VRC requires a highly coordinated recruitment of both viral and host factors, which optimise replication efficiency, stabilise membrane‐bound complexes, and facilitate template recognition (Li and Nagy 2011; Noueiry and Ahlquist 2003). Host proteins are known to play diverse roles in viral replication; for instance, the eukaryotic elongation factor 1A (eEF1A) enhances tomato bushy stunt virus replication by directly interacting with both the viral p33 replication protein and the 3′ UTR of the viral RNA, facilitating efficient replication (Li et al. 2009). Similarly, our in vitro RdRp assays demonstrated that recombinant NbHDR enhanced BaMV replication by promoting the synthesis of (+) gRNA (Figure 7d). While NbHDR does not appear to bind viral RNA directly, its interaction with BaMV replicase suggests that it may function as a replication cofactor. One possibility is that NbHDR improves BaMV replicase's recognition of the Ba‐77 promoter sequence, thereby enhancing (+) RNA synthesis. A comparable mechanism has been observed in influenza virus replication, where the acidic nuclear phosphoprotein 32 (ANP32) family serves as a host cofactor that bridges the polymerase subunits to enhance viral RNA synthesis (Zhu et al. 2023). Given the crucial role of host factors in shaping viral replication efficiency, future studies should investigate whether NbHDR modulates BaMV replicase function via conformational changes or acts as a molecular scaffold to facilitate template recognition. Furthermore, although the current results demonstrate a direct and functional involvement of NbHDR in BaMV replication, future research is necessary to explore whether additional metabolites derived from the MEP pathway, particularly other isoprenoid intermediates, also contribute significantly to this process. Addressing these gaps will provide deeper insights into how viruses hijack chloroplast‐associated pathways and may reveal novel strategies for engineering plant resistance against viral infections.

## 4 Experimental Procedures

### 4.1 Plant Materials and Growth Conditions

*Nicotiana benthamiana* plants were grown in a greenhouse maintained at 28°C, with a photoperiod of 16 h light and 8 h darkness.

### 4.2 Plant Inoculation by Agroinfiltration

Plasmids pKn (empty vector) (Liou et al. 2014), pKB‐RepHA21 (Huang, Hu, et al. 2017), and pKBG (Prasanth et al. 2011) were electroporated into *Agrobacterium tumefaciens* GV3850. Bacterial cultures were centrifuged, resuspended in infiltration buffer (10 mM MES, pH 5.5, 10 mM MgCl₂, 200 μM acetosyringone), adjusted to OD_600_ = 0.1, and infiltrated into the third and fourth leaves of 30‐day‐old *N. benthamiana* using a needleless syringe.

### 4.3 Preparation of Detergent‐Solubilised Membrane Fractions From *N. benthamiana* Tissues

The detergent‐solubilised membrane fractions were prepared from *N. benthamiana* leaves infected with pKn or pKB‐RepHA21 at 4 dpi, following a previously described method (Huang, Hu, et al. 2017). Briefly, 20 g of infected leaves were ground in liquid nitrogen and mixed with Buffer A (50 mM Tris–HCl, pH 7.6; 15 mM MgCl₂; 120 mM KCl; 20% glycerol; 0.1% β‐mercaptoethanol; 0.1 mM PMSF; and 0.1% protease inhibitor cocktail [Roche]). The homogenate was filtered, centrifuged at 500*g* for 10 min, and the supernatant was further centrifuged at 30,000 *g* for 30 min. The resulting pellet was resuspended in Buffer B (50 mM Tris–HCl, pH 8.2; 10 mM MgCl₂; 1 mM dithiothreitol [DTT]; and 0.1% protease inhibitor) and layered onto a 20%–60% sucrose gradient in Buffer C (50 mM Tris–HCl, pH 7.5; 100 mM NaCl; 3 mM MgCl₂; 2 mM DTT). After centrifugation at 72,100 *g* for 7.2 h, 10 fractions were collected. Fractions 5–7 (F567), containing the highest RdRp activity, were pooled and treated with 0.5% Sarkosyl to solubilise membrane‐bound RdRp. The mixture was centrifuged at 100,000 *g* for 30 min, and the supernatant was designated as the detergent‐solubilised membrane fractions.

### 4.4 IP‐MS

Immunoprecipitation was performed to identify host proteins associated with BaMV replicase complexes. The fraction of purified detergent‐solubilised membrane fractions (1 mL) was precleared with Protein A‐Sepharose CL‐4B (GE Healthcare) at 4°C for 1.5 h, followed by centrifugation at 3000 *g* for 2 min. The precleared sample (‘input’) was then incubated with 5 μL of anti‐HA antibody (Sigma‐Aldrich) for 4 h at 4°C, followed by the addition of 20 μL of protein A magnetic beads (GE Healthcare) and further incubation for 2 h. After washing, bound proteins were eluted, separated by SDS‐PAGE, and visualised by silver staining. Distinct protein bands were excised for LC–MS/MS analysis, and data were processed using Mascot (Matrix Science, v. 2.6.0) against the NCBI non‐redundant green plant database and the BaMV genome dataset.

### 4.5 Confocal Microscopy and Fluorescence Staining

To analyse the subcellular localisation of NbHDR in *N. benthamiana* protoplasts, the plasmid pEHDR‐OFP was constructed by amplifying the NbHDR coding sequence from *N. benthamiana* cDNA using primers XbaI/NbHDR_F (5′‐CGTCTAGAATGGCTATTCCTCTCCA‐3′) and KpnI/NbHDR_R (5′‐GCGGTACCGGCCAATTGTAAGGCTT‐3′). The purified PCR product was digested with XbaI and KpnI and ligated into the pEpyon‐OFP vector (Huang, Hu, et al. 2017) to generate pEHDR‐OFP.

For infiltration, *A. tumefaciens* cultures carrying pEHDR‐OFP and the chloroplast stroma marker pt‐gk (Nelson et al. 2007) were co‐infiltrated with cultures carrying pKn or pKB (Liou et al. 2014) into *N. benthamiana* leaves. At 3 dpi, protoplasts were isolated, and fluorescence images were captured using a confocal laser scanning microscope (FV3000; Olympus). Excitation wavelengths of 488, 561 and 640 nm were used to visualise GFP, OFP and chloroplast autofluorescence, respectively.

### 4.6 Co‐IP and Pull‐Down Assay

To investigate the interaction between BaMV replicase and NbHDR, *A. tumefaciens* cultures carrying pERep‐HA (Lee et al. 2019) or pEHDR‐OFP were adjusted to an OD_600_ of 0.5 and infiltrated into *N. benthamiana* leaves. At 3 dpi, infiltrated leaves were harvested, and total protein extracts were prepared. For Co‐IP, extracts were incubated with HA monoclonal antibody at 4°C for 4 h, followed by incubation with 50 μL of protein A magnetic beads (GE Healthcare) in binding buffer (50 mM Tris–HCl, pH 7.5; 3 mM MgCl_2_; 150 mM NaCl) at 4°C for 2 h. The beads were washed three times with 500 μL of binding buffer containing 1.5% NP‐40. After the final wash, the supernatants were removed, and sample buffer was added. The samples were boiled for 5 min and subjected to SDS‐PAGE and western blot analysis.

For the OFP pull‐down assay, 0.2 g of infiltrated leaves were homogenised in 600 μL of pull‐down (PD) extraction buffer (20 mM Tris–HCl, pH 7.5; 2 mM MgCl_2_; 150 mM NaCl; 5 mM DTT; 0.5% NP‐40; 4% protease inhibitor) and centrifuged at 4000 *g* for 10 min at 4°C. The supernatant was collected and incubated with 20 μL of RFP‐Trap magnetic beads (ChromoTek) at 4°C for 2 h, followed by three washes with 500 μL of PD extraction buffer containing 1.5% NP‐40. After washing, the supernatants were discarded, and sample buffer was added to the beads. The samples were boiled for 5 min and analysed by SDS‐PAGE and western blotting.

### 4.7 Y2H Assay

The coding region of NbHDR was amplified using primers SacI‐HDR CDSF and NotI‐HDR CDSR, digested with SacI and NotI, and ligated into pYESTrp and pHybLex vectors (Thermo Fisher Scientific) to generate pYESTrp‐NbHDR and pHybLex‐NbHDR. *Saccharomyces cerevisiae* L40 cells were transformed with a DNA mixture containing pHybLex‐ and pYESTrp‐based plasmids using the lithium acetate/PEG‐3350/TE method. After heat shock at 42°C with 10% dimethyl sulphoxide (DMSO), cells were plated on −Trp/Zeocin selection medium, followed by screening on −Trp/−His/Zeocin plates to assess protein–protein interactions. Positive and negative controls included pHybLex/Zeo‐Fos with pYESTrp‐Jun and pHybLex/Zeo‐Lamin with pYESTrp‐Jun, respectively.

### 4.8 VIGS

To knockdown *NbHDR* expression, a 389‐bp fragment of its 3′ UTR was amplified, digested with EcoRI and BamHI, and cloned into pTRV2 to generate pTRV2‐NbHDR. The construct was introduced into *A. tumefaciens* C58C1. *A. tumefaciens* cultures carrying pTRV2‐Luc, pTRV2‐NbKS (Huang et al. 2022), or pTRV2‐NbHDR were mixed 1:1 with *A. tumefaciens* containing pTRV1 (OD_600_ = 0.5) and co‐infiltrated into three leaves of *N. benthamiana*.

At 7 dpi, upper leaves were collected for RNA extraction using TriPure isolation reagent (Roche Life Science). Knockdown efficiency was assessed via RT‐qPCR using gene‐specific primers (NbKS_F: 5′‐GCAACACCCCGAATA‐3′ and NbKS_R: 5′‐ACTACGCTGCCCTCT‐3′ for *NbKS*; NbHDR/qPCRF: 5′‐CTCAAGCAACACTTCACATCTTCA‐3′ and NbHDR/qPCRR: 5′‐GGTAAGAAGTTCTCTTTCTCGACC‐3′ for *NbHDR*), with *actin* as an internal control (actin‐F: 5′‐GATGAAGATACTCACAGAAAGA‐3′ and actin‐R: 5′‐GTGGTTTCATGAATGCCAGCA‐3′). The third leaf above the infiltrated area was mechanically inoculated with 100 ng of BaMV virions in 10 μL of carborundum‐containing water. For GA treatment, a 200 μM GA solution was sprayed on the leaves 12 h before BaMV inoculation. RNA and protein were extracted at 3 dpi for further analysis.

### 4.9 Protoplast Isolation and BaMV Viral RNA Inoculation

Protoplasts were isolated from *N. benthamiana* leaves as described by Cheng and Tsai (1999). For viral RNA inoculation, 0.5 μg of BaMV RNA was introduced into 2 × 10^5^ protoplasts, followed by incubation at 25°C for 24 h. Total RNA and protein were then extracted for further analysis.

### 4.10 Northern Blot Assay

Total RNA was extracted from BaMV‐ or PVX‐inoculated leaves and protoplasts using TriPure isolation reagent (Roche Life Science). RNA samples were separated by electrophoresis, transferred onto nylon membranes (Hybond‐N+; GE Healthcare), and hybridised with ^32^P‐labelled riboprobes specific to BaMV or PVX RNA. BaMV probes were synthesised by transcribing HindIII‐linearised pBaHB with SP6 polymerase (Lin et al. 1993), while PVX probes were generated by transcribing HindIII‐linearised pPVXHE with T7 RNA polymerase in the presence of [α‐^32^P] UTP (Huang et al. 2012). Signals were detected using a phosphorimager (Fujifilm BAS 1500).

### 4.11 Protein Analysis

Total protein was extracted from leaves and separated on a 12% SDS‐PAGE gel. Proteins were transferred onto a PVDF membrane (Millipore) and probed with rabbit‐derived primary antisera against BaMV CP, PVX CP, NbHDR, OFP or T7 (1:5000 dilution).

### 4.12 Overexpression of *NbHDR*

The *NbHDR*‐T7 coding sequence was cloned into the pEp32K vector to generate pENbHDR‐T7 for transient expression in *N. benthamiana*. The plasmid was introduced into *A. tumefaciens* GV3850 via electroporation. Cultures were adjusted to an OD_600_ of 0.5 and infiltrated into *N. benthamiana* leaves using a needleless syringe.

To construct pEmNbHDR‐T7, a megaprimer was synthesised by a first PCR amplification using pENbHDR‐T7 as the template, with the primer pairs for mHDR‐F1 (5′‐GGTTGGTGGTTGGAACGCAAGCGCCACTTCACATCTTCAG‐3′) and SacI/NbHDR‐T7_R (5′‐GCGAGCTCTTACCCATTTGCTGTCCACCAGTCATGCTAGCCATGGCCAATTGTAAGGCTTC‐3′). The purified megaprimer was used in the second PCR amplification with the primer HDR4mF2 (5′‐CCATGGTAAATATTCTGCTGAGGCGACTGTTGCGACTGC‐3′). The purified megaprimer was used in the third PCR amplification with the primer KpnI/NbHDR_F. The purified PCR products were digested with KpnI and SacI and cloned into the pEpyon expression vector.

### 4.13 Immunodepletion of NbHDR from BaMV RdRp Preparation and In Vitro RdRp Assay

To deplete NbHDR from the BaMV RdRp preparation, 0.5 mL of the detergent‐solubilised membrane fractions purified from *N. benthamiana* leaves infected with pKB‐RepHA21 was incubated with 5 μg of purified IgG from either pre‐immune or anti‐NbHDR antiserum. The mixture was rotated at 4°C for 4 h, followed by the addition of protein A magnetic beads (GE Healthcare) to precipitate antibody‐bound complexes. The immunodepleted samples were analysed by western blotting with NbHDR‐ and HA‐specific antibodies to confirm depletion efficiency before being used in the in vitro RdRp assay. For exogenous template activity analysis, the depleted detergent‐solubilised membrane fractions were treated with micrococcal nuclease to digest endogenous templates. The RNA templates Ba 3′UTR and Ba‐77, as previously described (Cheng et al. 2001; Lin et al. 2005), were used in the RdRp assay. The in vitro RdRp activity and RNase protection assays were conducted following established protocols, with the modification that 200 ng of transcript RNA was added to the reaction mixture.

### 4.14 Expression and Purification of Recombinant Protein Trx‐His‐HDR

The coding sequence of *NbHDR* was amplified from *N. benthamiana* cDNA using the primers KpnI/NbHDR_F (5′‐GCGGTACCATGGCTATTCCTCTCCAG‐3′) and SacI/NbHDR_R (5′‐GCGAGCTCTTAGGCCAATTGTAAGGCTTC‐3′). The purified PCR products were digested with KpnI and SacI and cloned into the pET32a expression vector to generate pET32a‐NbHDR. The recombinant plasmid and the empty pET32a vector (control) were transformed into *E. coli* BL21 cells. Protein expression was induced with 0.4 mM IPTG at 16°C, and the recombinant Trx‐His‐NbHDR and Trx‐His proteins were purified using Ni^2+^‐NTA affinity chromatography (GE Healthcare).

## Conflicts of Interest

The authors declare no conflicts of interest.

## Supporting information


**Figure S1.** Properties of host proteins identified through LC–MS/MS analysis. (A) Summary of host proteins identified from LC–MS/MS analysis of co‐purified BaMV replication complexes. The table includes the identified host proteins, their known cellular functions, subcellular localisation, molecular weights (kDa), and identification scores. (B) Sequence coverage of identified proteins, with the matching sequences highlighted in red. These alignments demonstrate the regions of identified peptides relative to the full‐length protein sequence.

**Figure S2.** Effect of *NbHDR* knockdown on *Nicotiana benthamiana* plants. (A) Phenotype of *NbHDR*‐knockdown (HDRi) and control (Luci) *N. benthamiana* plants at 10 days post‐inoculation (dpi). Severe photobleaching was observed in the upper leaves of *NbHDR*‐knockdown plants. (B) Relative NbHDR gene expression levels in the Luci and HDRi plants were quantified by reverse transcription‐quantitative PCR at 10 dpi. Data represent the mean ± SD obtained from three independent experiments, with four individual plants for each experiment.

**Figure S3.** Effect of *NbHDR* knockdown on potato virus X (PVX) accumulation in *Nicotiana benthamiana* plants. (A) Protein analysis of PVX accumulation in the inoculated leaves of control and *NbHDR*‐knockdown plants. Total proteins were separated by 12% SDS‐PAGE, stained with Coomassie blue (loading control), and immunoblotted with anti‐PVX CP and anti‐NbHDR antisera. (B) Northern blot analysis of PVX RNA in *NbHDR*‐knockdown plants. At 7 days post‐inoculation (dpi), the upper leaves of control and *NbHDR*‐knockdown plants were infiltrated with agrobacterium carrying pKPG (PVX‐GFP). Total RNA was extracted from infiltrated leaves at 3 dpi, and PVX accumulation was analysed using α ^32^P‐labelled RNA probe to detect PVX RNAs. Data represent the mean ± SD obtained from three independent experiments, with three individual plants for each experiment. PVX genomic RNA was quantified and normalised using 28S rRNA as the loading control.

**Figure S4.** Expression and purification of recombinant proteins Trx‐His_6_‐HDR and Trx‐His_6_. (A, B) Analysis of Trx‐His_6_ protein expression and purification by SDS‐PAGE. Total cell lysate (T), supernatant (S), flow‐through (FT), wash (W) and elution (E) samples were collected and analysed. Protein concentrations were quantified using bovine serum albumen (BSA) standards. (C, D) Analysis of Trx‐His_6_‐HDR protein expression and purification by SDS‐PAGE. Samples were collected as described for Trx proteins and quantified using BSA standards. IPTG induction was used to express recombinant proteins.

**Figure S5.** Effect of transient expression of NbHDR and catalytically inactive NbHDR (mNbHDR) on bamboo mosaic virus (BaMV) accumulation. (A) Western blot analysis of total protein extracted from *Nicotiana benthamiana* leaves transiently expressing NbHDR‐T7 or mNbHDR‐T7 at 3 days post‐inoculation (dpi). NbHDR‐T7 and mNbHDR‐T7 expression levels were detected using anti‐NbHDR and anti‐T7 antibodies. (B) Northern blot analysis of BaMV genomic RNA (gRNA) and subgenomic RNAs in leaves co‐infiltrated with BaMV and either NbHDR or mNbHDR. BaMV RNAs were detected using an α‐^32^P‐labelled riboprobe complementary to the BaMV 3′ untranslated region (UTR). Quantification of BaMV gRNA was normalised to 28S rRNA. Data represent the mean ± SD from three independent experiments, each using three individual plants per experiment.

## Data Availability

The data that support the findings of this study are available from the corresponding author upon reasonable request.
