# Supplementary material for: NbHDR, A Host Protein Involved in the MEP Pathway, Interacts With Bamboo Mosaic Virus Replicase and Enhances Viral Accumulation
Source: Mol Plant Pathol. 2025 Jun 24;26(6):e70099. doi: 10.1111/mpp.70099 (PMC12186863; doi:10.1111/mpp.70099)
Supplement: Supplementary file 1 — Figure S1. Properties of host proteins identified through LC–MS/MS analysis. (A) Summary of host proteins identified from LC–MS/MS analysis of co‐purified BaMV replication complexes. The table includes the identified host proteins, their known cellular functions, subcellular localisation, molecular weights (kDa), and identification scores. (B) Sequence coverage of identified proteins, with the matching sequences highlighted in red. These alignments demonstrate the regions of identified peptides relative to the full‐length protein sequence. [file MPP-26-e70099-s005.docx]

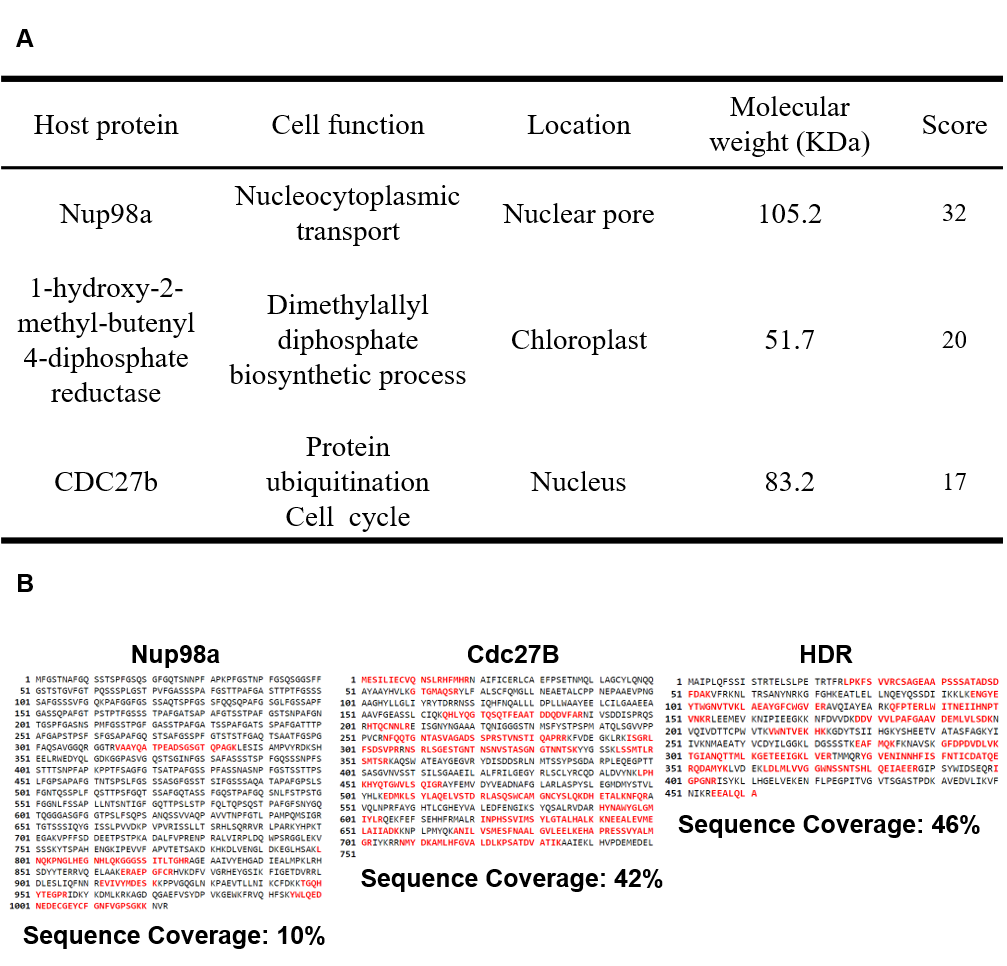


**Figure S1.** Properties of host proteins identified through LC-MS/MS analysis. (A) Summary of host proteins identified from LC-MS/MS analysis of co-purified BaMV replication complexes. The table includes the identified host proteins, their known cellular functions, subcellular localization, molecular weights (kDa), and identification scores. (B) Sequence coverage of identified proteins, with the matching sequences highlighted in red. These alignments demonstrate the regions of identified peptides relative to the full-length protein sequence.
