# Supplementary material for: NbHDR, A Host Protein Involved in the MEP Pathway, Interacts With Bamboo Mosaic Virus Replicase and Enhances Viral Accumulation
Source: Mol Plant Pathol. 2025 Jun 24;26(6):e70099. doi: 10.1111/mpp.70099 (PMC12186863; doi:10.1111/mpp.70099)
Supplement: Supplementary file 2 — Figure S2. Effect of NbHDR knockdown on Nicotiana benthamiana plants. (A) Phenotype of NbHDR‐knockdown (HDRi) and control (Luci) N. benthamiana plants at 10 days post‐inoculation (dpi). Severe photobleaching was observed in the upper leaves of NbHDR‐knockdown plants. (B) Relative NbHDR gene expression levels in the Luci and HDRi plants were quantified by reverse transcription‐quantitative PCR at 10 dpi. Data represent the mean ± SD obtained from three independent experiments, with four individual plants for each experiment. [file MPP-26-e70099-s003.docx]

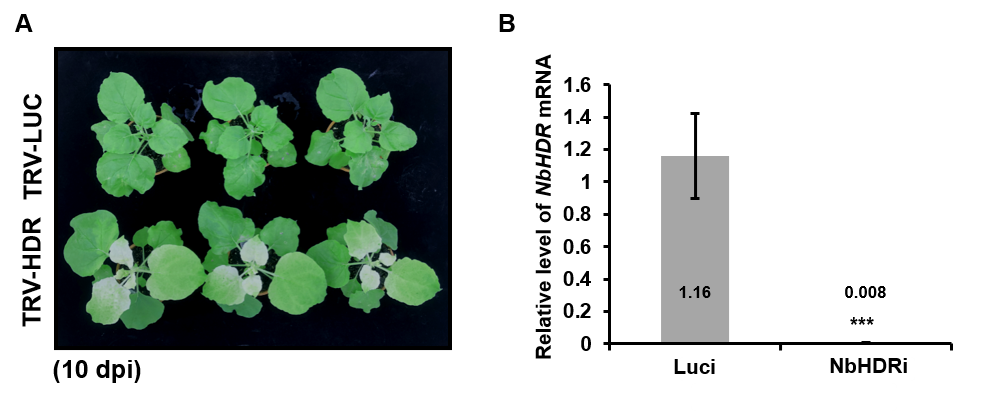


**Figure S2.** Effect of *NbHDR* knockdown on *N. benthamiana* plants. (A) Phenotype of *NbHDR*-knockdown (HDRi) and control (Luci) *N. benthamiana* plants at 10 dpi. Severe photobleaching was observed in the upper leaves of *NbHDR*-knockdown plants. (B) Relative NbHDR gene expression levels in the Luci and HDRi plants were quantified by RT-qPCR at 10 dpi. Data represent the mean ± SD of three independent experiments.
