# Supplementary material for: NbHDR, A Host Protein Involved in the MEP Pathway, Interacts With Bamboo Mosaic Virus Replicase and Enhances Viral Accumulation
Source: Mol Plant Pathol. 2025 Jun 24;26(6):e70099. doi: 10.1111/mpp.70099 (PMC12186863; doi:10.1111/mpp.70099)
Supplement: Supplementary file 3 — Figure S3. Effect of NbHDR knockdown on potato virus X (PVX) accumulation in Nicotiana benthamiana plants. (A) Protein analysis of PVX accumulation in the inoculated leaves of control and NbHDR‐knockdown plants. Total proteins were separated by 12% SDS‐PAGE, stained with Coomassie blue (loading control), and immunoblotted with anti‐PVX CP and anti‐NbHDR antisera. (B) Northern blot analysis of PVX RNA in NbHDR‐knockdown plants. At 7 days post‐inoculation (dpi), the upper leaves of control and NbHDR‐knockdown plants were infiltrated with agrobacterium carrying pKPG (PVX‐GFP). Total RNA was extracted from infiltrated leaves at 3 dpi, and PVX accumulation was analysed using α 32P‐labelled RNA probe to detect PVX RNAs. Data represent the mean ± SD obtained from three independent experiments, with three individual plants for each experiment. PVX genomic RNA was quantified and normalised using 28S rRNA as the loading control. [file MPP-26-e70099-s002.docx]

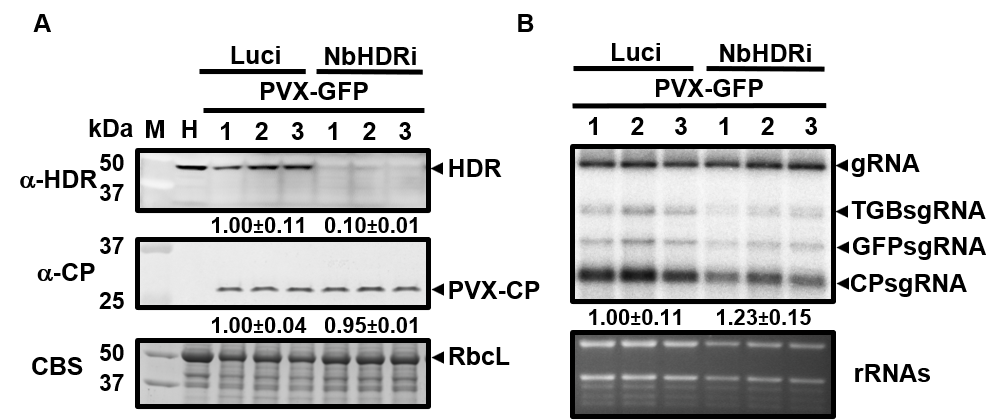


**Figure S3.** Effect of *NbHDR* knockdown on PVX accumulation in *N. benthamiana* plants. (A) Protein analysis of PVX accumulation in the inoculated leaves of control and *NbHDR*-knockdown plants. Total proteins were separated by 12% SDS-PAGE, stained with Coomassie blue (loading control), and immunoblotted with anti-PVX CP and anti-NbHDR antisera. (B) Northern blot analysis of PVX RNA in *NbHDR*-knockdown plants. At 7 dpi, the upper leaves of control and NbHDR-knockdown plants were infiltrated with agrobacterium carrying pKPG (PVX-GFP). Total RNA was extracted from infiltrated leaves at 3 dpi, and PVX accumulation was analyzed using α ³²P-labeled RNA probe to detect PVX RNAs. Data represent the mean ± SD from three independent experiments.
