# Supplementary material for: NbHDR, A Host Protein Involved in the MEP Pathway, Interacts With Bamboo Mosaic Virus Replicase and Enhances Viral Accumulation
Source: Mol Plant Pathol. 2025 Jun 24;26(6):e70099. doi: 10.1111/mpp.70099 (PMC12186863; doi:10.1111/mpp.70099)
Supplement: Supplementary file 4 — Figure S4. Expression and purification of recombinant proteins Trx‐His6‐HDR and Trx‐His6. (A, B) Analysis of Trx‐His6 protein expression and purification by SDS‐PAGE. Total cell lysate (T), supernatant (S), flow‐through (FT), wash (W) and elution (E) samples were collected and analysed. Protein concentrations were quantified using bovine serum albumen (BSA) standards. (C, D) Analysis of Trx‐His6‐HDR protein expression and purification by SDS‐PAGE. Samples were collected as described for Trx proteins and quantified using BSA standards. IPTG induction was used to express recombinant proteins. [file MPP-26-e70099-s001.docx]

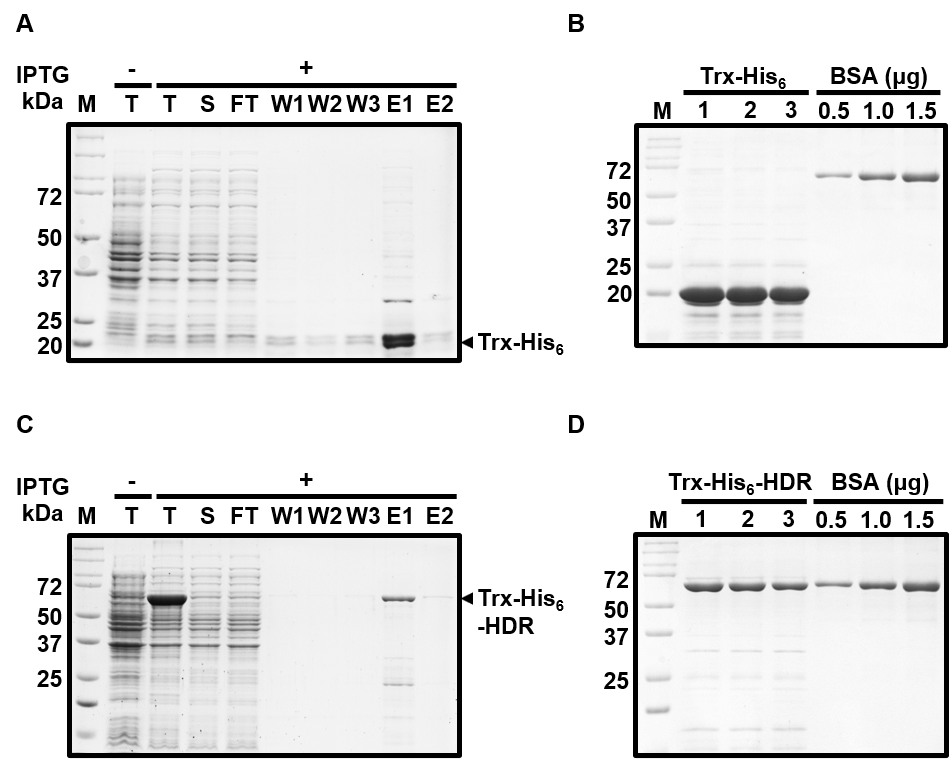


**Figure S4.** Expression and purification of recombinant proteins Trx-His_6_-HDR and Trx-His_6_. (A, B) Analysis of Trx-His_6_ protein expression and purification by SDS-PAGE. Total cell lysate (T), supernatant (S), flow-through (FT), wash (W), and elution (E) samples were collected and analyzed. Protein concentrations were quantified using BSA standards. (C, D) Analysis of Trx-His_6_-HDR protein expression and purification by SDS-PAGE. Samples were collected as described for Trx proteins and quantified using BSA standards. IPTG induction was used to express recombinant proteins.
