# Supplementary material for: NbHDR, A Host Protein Involved in the MEP Pathway, Interacts With Bamboo Mosaic Virus Replicase and Enhances Viral Accumulation
Source: Mol Plant Pathol. 2025 Jun 24;26(6):e70099. doi: 10.1111/mpp.70099 (PMC12186863; doi:10.1111/mpp.70099)
Supplement: Supplementary file 5 — Figure S5. Effect of transient expression of NbHDR and catalytically inactive NbHDR (mNbHDR) on bamboo mosaic virus (BaMV) accumulation. (A) Western blot analysis of total protein extracted from Nicotiana benthamiana leaves transiently expressing NbHDR‐T7 or mNbHDR‐T7 at 3 days post‐inoculation (dpi). NbHDR‐T7 and mNbHDR‐T7 expression levels were detected using anti‐NbHDR and anti‐T7 antibodies. (B) Northern blot analysis of BaMV genomic RNA (gRNA) and subgenomic RNAs in leaves co‐infiltrated with BaMV and either NbHDR or mNbHDR. BaMV RNAs were detected using an α‐32P‐labelled riboprobe complementary to the BaMV 3′ untranslated region (UTR). Quantification of BaMV gRNA was normalised to 28S rRNA. Data represent the mean ± SD from three independent experiments, each using three individual plants per experiment. [file MPP-26-e70099-s004.docx]

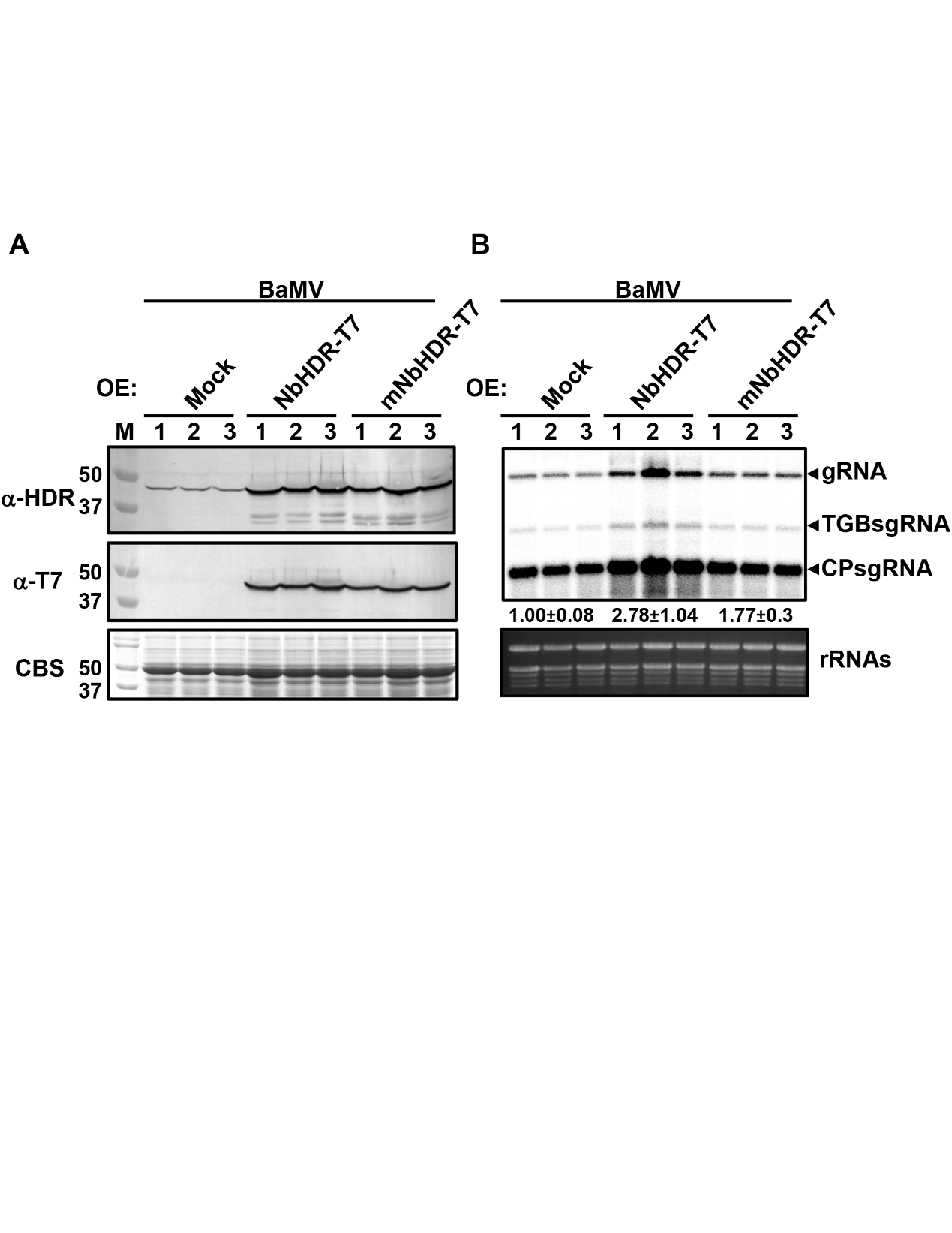


**Figure S5.** Effect of transient expression of NbHDR and catalytically inactive NbHDR (mNbHDR) on BaMV accumulation. (A) Western blot analysis of total protein extracted from *N. benthamiana* leaves transiently expressing NbHDR-T7 or mNbHDR-T7 at 3 dpi. NbHDR-T7 and mNbHDR-T7 expression levels were detected using anti-NbHDR and anti-T7 antibodies. (B) Northern blot analysis of BaMV genomic RNA (gRNA) and subgenomic RNAs in leaves co-infiltrated with BaMV and either NbHDR or mNbHDR. BaMV RNAs were detected using an α-³²P-labeled riboprobe complementary to the BaMV 3’-UTR. Quantification of BaMV gRNA was normalized to 28S rRNA. Data represent the mean ± SD from three independent experiments, each using three individual plants per experiment.
